# Comparison of the Postprocedural Quality of Life between Coronary Artery Bypass Graft Surgery and Percutaneous Coronary Intervention: A Systematic Review

**DOI:** 10.1155/2016/7842514

**Published:** 2016-02-18

**Authors:** Kaneez Fatima, Mohammad Yousuf-ul-Islam, Mehreen Ansari, Faizan Imran Bawany, Muhammad Shahzeb Khan, Akash Khetpal, Neelam Khetpal, Muhammad Nawaz Lashari, Mohammad Hussham Arshad, Raamish Bin Amir, Hoshang Rustom Kakalia, Qaiser Hasan Zaidi, Sharmeen Kamran Mian, Bahram Kazani

**Affiliations:** ^1^MBBS-Dow University of Health Sciences (DUHS), Karachi 74100, Pakistan; ^2^Kharadar General Hospital, Karachi, Pakistan; ^3^Cardiology, Civil Hospital, DUHS, Karachi 74100, Pakistan; ^4^Aga Khan University of Health Sciences, Karachi 74800, Pakistan; ^5^Department of Biological Sciences, The Lyceum, Karachi 75600, Pakistan; ^6^The Karachi Grammar School, Karachi 75600, Pakistan

## Abstract

The treatment of choice between coronary artery bypass graft surgery (CABG) and percutaneous coronary intervention (PCI) has remained unclear. Considering quality of life (QOL) increases life expectancy, we believe QOL should be important in determining the optimum treatment. Thus the objective of this review was to illustrate the comparative effects of CABG and PCI on postprocedural QOL.* Methods.* We searched PubMed (Medline) and Embase from inception of the databases to May 2014 using “PCI versus CABG quality of life”, “Percutaneous Coronary intervention versus Coronary artery bypass graft surgery Quality of life”, “PCI versus CABG health status”, “Angioplasty versus CABG”, “Percutaneous coronary intervention versus coronary artery bypass surgery health status”, and different combinations of the above terms. 447 articles were found. After applying strict exclusion criteria, we included 13 studies in this review.* Results.* From the 9 studies that compared QOL scores at 6 months after procedure, 5 studies reported CABG to be superior. From the 10 studies that compared QOL among patients at 1 year after procedure, 9 reported CABG to be superior.* Conclusion.* It can be established that CABG is superior to PCI in improving patient's QOL with respect to all scales used to determine quality of life.

## 1. Introduction

Coronary artery disease (CAD) is one of the leading causes of mortality and morbidity worldwide. Due to the high prevalence of CAD, both percutaneous coronary intervention (PCI) and coronary artery bypass graft surgery (CABG) are extremely common procedures [1]. Both techniques have proven to be safe and effective in treating CAD. Several studies have shown that both CABG and PCI can improve mortality and quality of life (QOL) significantly [2]. However the treatment of choice between the two in certain commonly witnessed clinical scenarios such as unprotected left main CAD has remained uncertain for a long time.

Many randomized clinical trials have been conducted to compare the rates of mortality and myocardial infarction among CABG and PCI patients. Apart from the few studies [3, 4] that have established that CABG causes significant reduction in long term mortality and myocardial infarctions, many trials have demonstrated little difference [5–9]. We believe that in addition to mortality, QOL can be an important factor in deciding patient management especially among the indications where gray zones occur. QOL is a vital outcome after any medical procedure and for many people QOL is of equal significance, if not more, to increasing life expectancy. QOL is clearly the primary goal and benefit of any treatment plan [10].

Despite the several studies comparing QOL after CABG versus angioplasty among patients with CAD, the results have remained unclear. Moreover, very few systemic reviews have focused on QOL in deciding the better choice of treatment. The primary objective of this systematic review is to provide a complete picture of the comparative effects of CABG and PCI on postprocedural QOL.

## 2. Methods

### 2.1. The Literature Search

An extensive literature search was conducted using PubMed and Embase to find all published original articles comparing QOL after PCI and CABG from inception of the databases till May 2014. “PCI versus CABG quality of life”, “Percutaneous Coronary intervention versus Coronary artery bypass graft surgery Quality of life”, “PCI versus CABG health status”, “Angioplasty versus CABG”, “Percutaneous coronary intervention versus coronary artery bypass surgery health status”, and different combinations of above terms were used for the retrieval of original articles. Due to inadequate resources, we had to limit our search parameters to English language only. The reference list of all the retrieved articles was also hand searched to spot any study that was missed during the database search.

### 2.2. Study Selection

**Figure 1:**
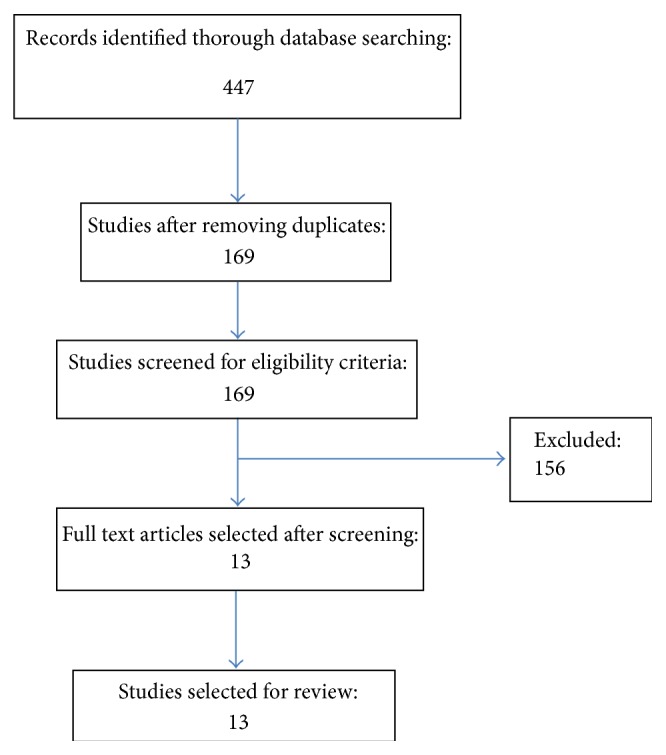
Showing the literature search results.

447 articles were found and subsequently reviewed by two authors in light of the exclusion criteria. Studies were excluded on the following basis (1) retrospective studies, (2) studies performed on animal models, (3) studies that were conducted without using a validated questionnaire to measure quality of life, (4) duplicate publication, (5) difficulty in extracting outcomes of interest from the study, and (6) studies which used minimally invasive coronary artery bypass graft surgery technique. Any dispute regarding exclusion of a study was settled by discussion and consensus. After deciding on the studies to be included, the data was extracted and verified by two authors. Considering the enormous heterogeneity, no attempt was made to pool the data. The selected studies were then summarized in a tabular form for easy reviewing (Table 2).  Figure 1 shows the results of the literature search and study selection.

## 3. Results

Out of the total of 447 articles retrieved, 13 studies were incorporated in our systemic review.  Table 1 shows the baseline characteristics of the patients from the included studies. From these 13 studies, 9 compared QOL scores at six months between CABG and PCI [1, 8, 11, 13–17, 20]. Out of these 9, 5 studies [1, 11, 13, 16, 20] reported that patients in the CABG group experienced better QOL six months after the procedure. The remaining four studies [8, 14, 15, 17] found QOL to be the same in both groups. Similarly, 10 studies [2, 8, 11–16, 18, 19] were found that compared QOL among the two sets of patients at 1 year after the procedure. From these, 9 studies [8, 11–16, 18, 19] stated that QOL scores were significantly higher in the CABG group at 1 year after procedure while the remaining one study [2] found the QOL scores to be the same in both groups.

## 4. Discussion

### 4.1. Seattle Angina Questionnaire (SAQ)

Five studies [11–15] were identified that had used SAQ for determining QOL. The SAQ is considered to be a superior assessor of clinical health status, with greater sensitivity and better interpretability than other health status scales, such as the SF-36. This scale is divided into 5 domains, namely, physical limitation score, angina frequency score, quality of life score, treatment satisfaction score, and angina stability score.

### 4.2. Physical Limitation

Borkon et al. reported that patients who had undergone CABG were able to achieve superior physical function one year after procedure as compared to the PCI patients, even though it had declined one month after revascularization. This decline was also reported by Cohen et al., with no significant differences at 6 months follow-up. Abdallah et al. found similar results as well, with physical function superiority by CABG following into the 2nd year of follow-up as well.

Another important finding by Borkon et al. was the effect of restenosis on physical function. Patients with restenosis reported a significant decrement in physical tasks, which at one month after procedure was comparable in degree to that observed in patients who had undergone CABG. Moreover, in spite of revascularization for restenosis, physical function score did not increase to the same degree as that observed in patients who had undergone fruitful PCI or CABG. Therefore, functional limitation in the PCI group was primarily due to the effect of restenosis. Zhang et al. however reported that because of repeat revascularization for restenosis in the PCI cohort, the degree of difference between the two groups decreased over time. Another finding worth mentioning was that the authors also studied the effect of repeat intervention on the relative benefit of CABG versus PCI. Average 6-month follow-up SAQ scores were low for those patients who required repeat intervention. The scores improved gradually between 6 and 12 months but did not increase to the same degree as that observed in patients who had undergone fruitful PCI or CABG.

### 4.3. Quality of Life

Borkon et al. showed that QOL score is significantly greater in the CABG group than in the PCI group at 6 and 12 months postoperatively (*p* < 0.0001), primarily due to restenosis in the PCI group. Repeat revascularization eventually increased the QOL score to the same level as that of patients who did not require repeat intervention, but less than that of CABG patients. In short, restenosis after PCI reduced the QOL scores over the 12-month period of study (*p* < 0.0001). Abdallah et al. had similar results as well in the QOL domain, with superior scores for CABG extending into 2-year follow-up.

Spertus et al. showed that CABG surgery resulted in greater 1-year QOL scores in patients with intermediate (*p* = 0.0004) and high (*p* = 0.006) risk of restenosis as compared to PCI. Similarly, Cohen et al. also demonstrated that QOL score was significantly higher in the CABG group than in the PCI group at 12 months (difference between groups = 2.4 points; *p* = 0.03).

### 4.4. Angina Frequency

Borkon et al. reported that CABG is associated with greater relief from angina than PCI (*p* < 0.001), primarily due to the effect of restenosis in the PCI group. Multivariable analysis confirmed the benefit of CABG over PCI (*p* = 0.004). The authors concluded that patients with restenosis had greater anginal distress over time than patients who underwent CABG or PCI without restenosis. Abdallah et al. reiterated the same results, with CABG associated with greater anginal relief as compared to PCI at 2-year follow-up (mean treatment benefit 1.3 [95% CI, 0.3–2.2], *p* < 0.01). Moreover, Spertus et al. concluded that CABG resulted in better anginal outcomes in patients with intermediate (difference in SAQ angina frequency scores favoring CABG = 6.1 ± 1.7 points, *p* = 0.0003) and high (SAQ angina frequency difference = 10.8 ± 4.2, *p* = 0.01) risk of restenosis as compared to PCI. Cohen et al. and Zhang et al. also found that CABG is associated with better anginal outcomes at both 6 and 12 months as compared to PCI.

Therefore, it can be safely concluded from these studies that CABG results in better physical function, QOL, and anginal outcomes during 1-year postoperative period in comparison to PCI. In addition, CABG yields in better anginal outcomes in patients with moderate or high risk for restenosis and restenosis is found to reduce QOL scores.

### 4.5. The Medical Outcomes Study Short Form 36 Health Survey

Five studies [15–19] in all were found to have used SF-36 in comparing the QOL between the two treatment procedures. The SF-36 is a generic written questionnaire comprising a total of 36 items that cover 8 health constructs: physical functioning, role limitations due to physical problems, bodily pain, energy and vitality, social functioning and role limitations due to emotional problems, and mental health. In the literature reviewed, two methods of reporting the results were found. In one form, the scores from these 8 original scales are aggregated into the Physical Component Summary (PCS) scores and the Mental Component Summary (MCS) scores reflecting the overall physical and mental health. In another form, the scores for individual items within each domain are summated and compared. We compare the effects of CABG and PCI in terms of physical and mental health.

### 4.6. Physical Health

Rumsfeld et al. investigated the health related quality of life (HRQL) outcomes in the Department of Veteran Affairs Angina with Extremely Serious Operative Mortality (AWESOME) study population at six months. No significant differences were reported at the end of the 6 months. However, diabetes was associated with a negative trend in PCS scores in the CABG surgery group. Furthermore diabetes, COPD, and elevated serum creatinine were identified as key predictors for worse physical health at six months.

Szygula et al. reported HRQL impairment in the PCS scores of the PCI group at 12 months in their investigation. This was attributed to worse outcomes in all four domains of physical health: physical functioning, role physical, bodily pain, and general health. A greater proportion of PCI patients were found to have unstable angina at twelve months, with substantially higher systolic and diastolic blood pressures. Similar to these results, the rate of repeat revascularization was also high in PCI over CABG surgery. It is worth mentioning here that a negative trend was found between systolic pressure and the PCS and MCS scores of only the PCI cohort.

Favarato et al. conducted a different study, in that they compared the effect of CABG surgery, PCI, and Medical Therapy (MT) on QOL in the study population of the Medicine, Angioplasty and Surgery Study (MASS-II) trial. At twelve months, a significant difference was found in the number of angina free patients with a higher proportion belonging to the CABG group (88%). In addition, only 0.5% of CABG patients were found to have undergone repeat revascularization as compared to a high number of PCI patients (13.3%). Overall, a positive trend in QOL assessment was found; though CABG showed worse scores at baseline in terms of physical functioning and vitality, it showed significant superiority over PCI and MT in vitality (*p* = 0.0024) and physical functioning (*p* = 0.0029) at six months. It is imperative to mention that no significant treatment difference was observed in any of the eight domains at twelve months. Though professional and occupational status (43.9% of the men were employed) was evaluated, no significant differences were reported based on choice of procedure at the end of the year. However, once the final scores were adjusted by their initial scores as covariable and other factors using a general linear model, it was observed that CABG was superior to PCI in terms of general health, a finding consistent with that of the Coronary Angioplasty versus Bypass Revascularization Investigation (CABRI) trial.

The Arterial Revascularization Therapy Study (ARTS) trial reported HRQL outcomes using the European QOL ED-5D at one and three years, but Domburg et al. also used the SF-36 survey and evaluated anginal status at baseline, 1, 6, 12, and 36 months. They made use of scale scores rather than the summary scores. At one month postop, similar to previous studies, a significant improvement was seen in the PCI group while the CABG patients showed a decrease in most SF-36 subscales. However from 1 month to 6 months, the CABG group showed a substantial increase. The only domain which showed a significant difference up till 6 months was bodily pain where PCI was superior to CABG, because of procedural effects. Between 6 and 12 months, the outcomes remained almost equal. Eventually three of the subdomain scores (physical functioning, social functioning, and general health) were substantially superior in CABG over PCI, where, with longer follow-up of 36 months, all scores decreased slightly to meet at the same level. A subgroup analysis showed that a greater proportion of PCI patients had angina at all times as compared to CABG. It is worth mentioning that this gap grew smaller with time. It is noteworthy that the prevalence of angina in those PCI patients who did not require another procedure is lesser than those who did but still greater than the CABG group. Furthermore, while diabetic PCI patients showed a decrease in general health scores at all-time points, physical functioning showed a steady decrease only with time.

The Synergy between PCI with Taxus and Cardiac Surgery (SYNTAX) trial differed from other studies because of its use of drug eluting stents in PCI. Like the ARTS study, it evaluated HRQL outcomes using EuroQOL ED-5D instrument and SF-36 survey, as well as SAQ. Similar to the findings of Favarato et al., Cohen et al. reported significant superiority of PCI over CABG in physical health status one month postop. However, except for role limitations because of physical problems, this difference did not persist at six months and, by twelve months, CABG gained significance over PCI in general health.

### 4.7. Mental Health

The AWESOME study yielded no significant difference in the MCS scores. As a result of the multivariable analyses, current smoking and hypertension were identified as worse outcome predictors at six months of mental health. The use of beta blockers was found to be associated with a better mental health outcome. Similarly, Szygula et al. reported no substantial difference in MCS scores between the two arms. With the MASS-II trial, Favarato et al. reported no significant difference in any of the three study arms in terms of mental and emotional functioning. However, a time effect was reported which showed an increase in these scores at six months postop in all three treatment arms which meant betterment of health. The authors commented that no significant pattern could be obtained because mental health poses a not so direct link to coronary artery disease. The ARTS and SYNTAX trials also yielded no significant correlations or differences between the two procedures in terms of mental health status.

These studies yielded vague results, possibly owing to the generic nature of the SF-36 that simply evaluates the general health of the patients and is not disease-specific, and the varied composition of the study pools is included. Where Rumsfeld et al. reported no difference at 6 months after procedure, all others reported superiority of CABG over PCI at 12 months after revascularization. A high rate of repeat procedures was also reported for the PCI cohort and the difference in treatment procedures was found to decrease over time.

In terms of mental health, all authors ascertained that no correlation can be determined.

### 4.8. Other Scales Discussion

Our extensive literature search also yielded further four studies [1, 2, 8, 20] that used instruments apart from SAQ and SF-36 to assess health related quality of life of patients belonging to either of the treatment groups for comparison.

Pocock et al. utilized the Nottingham Health Profile (NHP) to assess the perception of health amongst the study population of the Randomized Intervention Treatment of Angina (RITA) trial. For this study, improvement in health status was quite substantial for both cohorts at 6 months and 2 years as compared to preop days without any significant difference. It can be mentioned though that the CABG patients were found to be slightly superior in all six dimensions (energy, pain, emotional reactions, social isolation, sleep, and physical mobility) at 6 months (mean difference of 1.21 points). Pocock et al. also found a slightly increased prevalence of health problems in the PTCA arm of the study at both end points by averaging the total number of life aspects adversely affected over the 6-month and 2-year visits (means, 0.87 and 1.07 aspects for CABG and PTCA groups, resp.; difference, 0.22; 95% CI, 0.00 to 0.39; *p* = 0.05). In terms of anginal status, no difference between the two cohorts could be found.

Similarly, Währborg [2] investigated perception of health in the Coronary Angioplasty versus Bypass Revascularization Investigation (CABRI) trial study population but used 12 separate questions apart from the NHP. Overall betterment was reported for total life scores and separate dimension scores for both study groups, relative to baseline. These results were consistent with the findings of the RITA study. Währborg [2] also commented on the energy difference they found to be in favor of CABG patients in NHP part 1 as well as in item 1 of the 12 separate questions. No other study commented on this trend. The authors attributed this trend to the CABRI trial protocol allowing incomplete revascularization of the PTCA patients and not excluding those patients who had totally occluded vessels. With respect to anginal status, no association could be made.

Brorsson et al. evaluated QOL and functional status using multiple standardized questionnaires. 33.5% of angioplasty patients had to undergo a repeat revascularization procedure within a 2-year follow-up. At 4-year follow-up, no significant difference in survival was found. Additionally, the frequency of angina symptoms was markedly less in both study arms by 6 months, with a great improvement in the CABG group (only 11.5% reporting symptoms). However it was found that the number of angina free patients and those who had not used sublingual nitrates was greater in the PTCA cohort at 6 months, though patients who had not used sublingual nitrates for a month were greater in the CABG group at 48 months. It is worth mentioning that the relative superiority of CABG over PTCA showed a decreasing trend over the follow-up period. With further analyses, it was found that a preop history of high anginal frequency at baseline was a strong predictor of high anginal frequency at follow-up. CABG surgery and history of positive stress test were found to be associated with lower anginal frequency at 6 months.

They also used the Swedish QOL survey (SWED-QUAL) to assess the well-being of the patients. A strong correlation between anginal frequency and both physical functioning and health perception was found, while that of anginal frequency and emotional well-being and sleep was not noteworthy. Preop QOL was the most constant predictor of postop QOL at 6 and 48 months for all 5 life scales. CABG patients saw greater improvement in physical functioning and general health status at 6 months than the PTCA group, but this was not present at 48 months. Male patients showed greater improvement in physical functioning and pain relief than women. Increasing age was also associated with low levels of physical functioning by 48 months but had no effect on any other scales. No differences in degree of improvement were found on the basis of history of smoking, COPD, hypertension, or positive stress test. No difference was also found on the basis of diabetes mellitus.

Serruys et al. evaluated angina status and QOL, using the EuroQOL questionnaire, as a secondary objective of their randomized control trial. A greater proportion of patients in the CABG group were found to be angina free throughout the study period. The QOL assessment initially yielded favorable results for PCI at 1 month, but this gap was reduced at 6 months and eventually became slightly significant in favor of CABG at 12 months. Elevated level of CK-MB was the main outcome predictor in the surgery group and diabetes mellitus in the stent group.

## 5. Limitations

There are several limitations in our systematic review that need to be considered. Firstly, using only one database and applying English language restriction may have resulted in some pertinent studies not being included. Secondly, there was significant heterogeneity among the sample populations considered in our review. However, we feel that regardless of this heterogeneity, the general pattern is very clear as shown by our review. In the future, a meta-analysis on this topic might help to elucidate and confirm the pattern as shown.

## 6. Conclusion

In this review we have summarized the major studies pertaining to the comparison of postprocedural QOL between CABG and angioplasty. Our review suggests that although angioplasty, through its less invasive nature, might provide better QOL within the first few months, CABG is superior in providing improved QOL at both 6 and 12 months after procedure and in the long run.

## Figures and Tables

**Table 1:** Showing the studies included in the review.

Serial number	Author name/date	Scale	*N*	Baseline mean age	Males (% of patients)
			(CABG/PCI)	(CABG/PCI)	(CABG/PCI)
1	Zhang et al., 2003 [11]	SAQ	500/488	61.4/61.4	79.0/79.0
2	Spertus et al., 2005 [12]	SAQ	432/1027	66.0/66.1	74.0/70.0
3	Borkon et al., 2002 [13]	SAQ	223/252	67.0/64.0	66.0/68.0
4	Abdallah et al., 2013 [14]	SAQ	947/953	63.0/63.2	69.8/73.2
5	Cohen et al., 2011 [15]	SAQ and SF-36	897/903	65.0/65.2	78.9/76.4
6	van Domburg et al., 2008 [16]	SF-36	492/483	62.0/61.0	77.0/77.0
7	Rumsfeld et al., 2003 [17]	SF-36	196/193	67.3/67.6	98.5/98.9
8	Szygula-Jurkiewicz et al., 2005 [18]	Sf-36	104/392	62.4/61.8	71.2/66.3
9	Favarato et al., 2007 [19]	Sf-36	175/180	59.0/59.0	53.0/40.0
10	Währborg 1999 [2]	The Nottingham Health Profile	154	—	—
11	Pocock et al., 1996 [20]	Nottingham Health Profile	1011	—	—
12	Brorsson et al., 2001 [1]	Swedish Quality of Life Survey	252/349	62.8/59.8	77.8/75.1
13	Serruys et al., 2001 [8]	EuroQOL questionnaire	579/593	61.0/61.0	76.0/77.0

**Table 2:** A summary of the reviewed articles.

Serial number	Author name/date	Summary
1	Zhang et al., 2003 [11]	Quality of life scores were higher in patients opting for CABG at both 6 months and 1 year.

2	Spertus et al., 2005 [12]	1-year quality of life scores were significantly better for patients treated with CABG surgery as opposed to PCI.

3	Borkon et al., 2002 [13]	Patients undergoing CABG achieved greater quality of life at 6 and 12 months after their procedure.

4	Abdallah et al., 2013 [14]	For patients with diabetes and multivessel CAD, CABG surgery provided slightly better quality of life than PCI using drug-eluting stents. The magnitude of benefit was small, without consistent differences, beyond 2 years.

5	Cohen et al., 2011 [15]	Among patients with three-vessel or left main coronary artery disease, scores for quality of life were higher with PCI than with CABG, at 1 month. These differences were no longer apparent at 6 months. At 12 months, the score for quality of life was higher in the CABG group than in the PCI group.

6	van Domburg et al., 2008 [16]	Both stenting and CABG resulted in significant improvement in QOL of patients, up to one year, with CABG patients showing greater improvements.

7	Rumsfeld et al., 2003 [17]	High-risk patients with medically refractory ischemia randomized to PCI versus CABG surgery have equivalent six-month quality of life.

8	Szygula-Jurkiewicz et al., 2005 [18]	There is a significant difference in health-related quality of life, 12 months after percutaneous coronary intervention and coronary artery bypass graft surgery with the difference favoring the patients undergoing bypass.

9	Favarato et al., 2007 [19]	After 1 year of follow-up, the patients submitted to CABG were the ones that presented the greater improvement in QOL.

10	Währborg 1999 [2]	This study has shown that there is no general difference in health-related quality of life 1 year after bypass surgery or angioplasty.

11	Pocock et al., 1996 [20]	Both intervention strategies produce similar benefits for quality of life over several years.

12	Brorsson et al., 2001 [1]	Both bypass surgery and angioplasty lead to improved quality of life for patients with chronic stable angina and one- or two-vessel coronary artery disease. Bypass surgery is associated with better quality of life at 6 months, but by 48 months quality of life is similar for both sets of patients.

13	Serruys et al., 2001 [8]	A significantly better quality of life was reported with stenting, as compared to bypass surgery, after 1 month. No differences were reported between the two groups at 6 months and a slight difference in favor of surgery was found after 12 months.
